# Implementing in hospital technology‐assisted mobility initiatives: A scoping review

**DOI:** 10.1002/jhm.70262

**Published:** 2026-01-25

**Authors:** Pamela Mathura, Amy Wenzel, Liz Dennett, Narmin Kassam

**Affiliations:** ^1^ Department of Medicine University of Alberta Edmonton Alberta Canada; ^2^ Alberta Health Services Edmonton Alberta Canada; ^3^ Faculty of Kinesiology, Sport, and Recreation University of Alberta Edmonton Alberta Canada; ^4^ Geoffrey and Robyn Sperber Health Sciences Library University of Alberta Edmonton Alberta Canada

## Abstract

**Background:**

Hospitalized patients are typically inactive, though evidence highlights the effectiveness of mobility‐enhancing interventions in improving health outcomes. Technology‐assisted approaches are increasingly used to encourage patient movement.

**Objectives:**

This scoping review examines technology‐assisted initiatives designed to promote physical activity in hospitalized adults and explores implementation strategies used to facilitate these initiatives.

**Methods:**

The Arksey and O'Malley's 2005 framework was used. Studies were identified through searches of MEDLINE, EMBASE, CINAHL, Scopus, and Cochrane trials. Bibliographies of included studies were searched. Characteristics of technology‐assisted interventions and implementation strategies used were extracted, categorized, and analyzed for frequency.

**Results:**

Thirty papers representing 28 unique initiatives were identified from 6049 articles. The technology used were wearable step or activity counters (20), exergames (6), mobile ambulation reminders (3), and applications for in‐bed exercises (1). Five implementation strategies reported from three studies were coded using the Expert Recommendations for Implementing Change: identifying and preparing champions, facilitating relay of clinical data, conducting educational meetings, developing and distributing educational materials. Eight behavior change techniques were reported: encouragement, collaborative goal setting, increasing daily goals, progress tracking, visual data display, patient education, environmental modification and physical therapist support.

**Conclusion:**

The implementation of technology‐assisted mobility interventions in hospitals to enhance patient mobility is emerging. Applying implementation and behavioral science frameworks may enhance effectiveness. Future studies are required to evaluate implementation strategy outcomes and to examine patient and clinician experiences to inform intervention adaptation and to facilitate integration into routine clinical hospital ward/unit practice.

## INTRODUCTION

Hospitalized adults are usually inactive and spend most of their time in bed during their stay, which is associated with increased mortality, functional decline, and cognitive impairment.^1^ Research indicates that patients who move more during their hospital stay have better outcomes.^2^, ^3^ Initiatives promoting mobility and progressive physical activity during hospitalization are increasing and may be implemented by a variety of healthcare providers (i.e., nurses, physical therapists, etc.), family members, and/or patients.^1^ The possible benefits of mobility initiatives include improved physical function, reduced length of stay, and 30‐day readmissions, but research integrating these initiatives into hospital processes is lacking.^4^

A recent review of enablers and barriers to implementing mobility initiatives indicated that many factors influenced the mobility behavior of hospitalized patients, such as staffing, equipment, and health care providers′ lack of time.^3^ Developing and implementing multicomponent initiatives may address these barriers to improve mobility behavior during hospitalization.^3^ The coronavirus disease (COVID‐19) pandemic accelerated the use of technology in medical practice^5^ resulting in the use of technology‐assisted mobility initiatives.^6^ These initiatives use technological devices to support ambulation such as exergames^7^, ^8^, ^9^ and wearable devices that monitor^10^, ^11^, ^12^ and/or prompt movement.^13^ We define an initiative as both the intervention components (technologies and accompanying behavior change techniques) and the implementation strategies. Behavior change techniques are reproducible pieces of interventions that are used to facilitate changes in behavior.^14^ Implementation strategies are, in this context, methods or techniques used to enhance the adoption, implementation, and sustainability of a technology‐assisted mobility intervention.^15^

While these initiatives show promise, there is a lack of synthesized evidence on which technologies, behavior change techniques, and implementation strategies are most effective and why. As the outcomes of these initiatives are variable,^6^ understanding which specific components contribute to success is important for furthering research in this field.^2^ As the first step to address this gap in knowledge, this scoping review was conducted to summarize the published evidence on technology‐assisted mobility initiatives in a hospital setting, identifying the types of technology, behavior change techniques, and the implementation strategies reported.

## RESEARCH QUESTION AND PURPOSE

The review questions were: *1. What are the technology‐assisted initiatives used to promote physical activity for adult patients in the acute care/hospital setting? and 2. What implementation strategies were used to support uptake of these interventions?* The purpose was to examine the variety of technology‐assisted devices and interventions used in hospitals and implemented by any clinical staff. The objectives were to describe and present an overview of hospital‐based technology‐assisted initiatives identified in published literature and to identify the implementation strategies used to incorporate these initiatives into practice. The importance of implementation science in clinical practice is well established^16^ and to the best of our knowledge, no review of mobility interventions has yet considered implementation. This summary provides health organizations and medical leaders with information that can be used to establish mobility initiatives for hospitalized patients. Additionally, we intend to identify gaps in the literature to aid the planning and commissioning of future quality improvement and implementation research.

## METHODS

### Study design

No protocol was published for this review. This scoping review was conducted using the Arksey and O′Malley methodological framework^17^ and reported in accordance with the Preferred Reporting Items for Systematic Reviews and Meta‐Analyses Extension for Scoping Reviews.^18^ Ethical approval was not required for this review.

### Eligibility

Included studies needed to describe a technology‐assisted mobility intervention in a hospital setting.

#### Population

The majority of patients enrolled in the included studies must be adult inpatients.

#### Concepts

Terminology for mobility is inconsistent^2^ and for this review, mobility will be defined as the quantity of movement ranging from in‐bed movements to light physical activity (activity with an intensity between 1.5 and 3 metabolic equivalents).^19^ For the purposes of this study, a mobility intervention is defined as an intervention that aims to promote patient mobility, while technology‐assisted mobility interventions are defined as mobility interventions using technology of any kind to promote movement. This does not include interventions where technology is only used to measure movement. A technology‐assisted mobility initiative includes the technology, the behavior change techniques used, and the implementation strategies applied.

#### Contexts

Studies must be conducted in hospital settings. We excluded studies where the intervention lasted over 14 days, took place in a rehabilitation hospital, an ICU, or a maternity ward, involved neuromuscular electrical stimulation, or aimed at mobility education, general health, or preoperative care (File S1, Section 2). Studies conducted in rehabilitation hospitals were excluded because these settings prioritize restoring function, mobility, and independence through specialized teams, which present unique barriers and enablers to promoting physical mobility. Interventions lasting over 14 days were excluded to focus on hospital settings with high patient turnover, such as general internal medicine units, where the length of stay is typically under 14 days for most patients.^20^

#### Sources of evidence

This review included all study designs, trial registrations, and protocols (if full‐length studies were unavailable or unpublished) from any country. We excluded conference abstracts, reviews, editorials, unpublished studies, and non‐English articles that could not be translated using Google Translate.

### Search strategy and article selection

**Figure 1:**
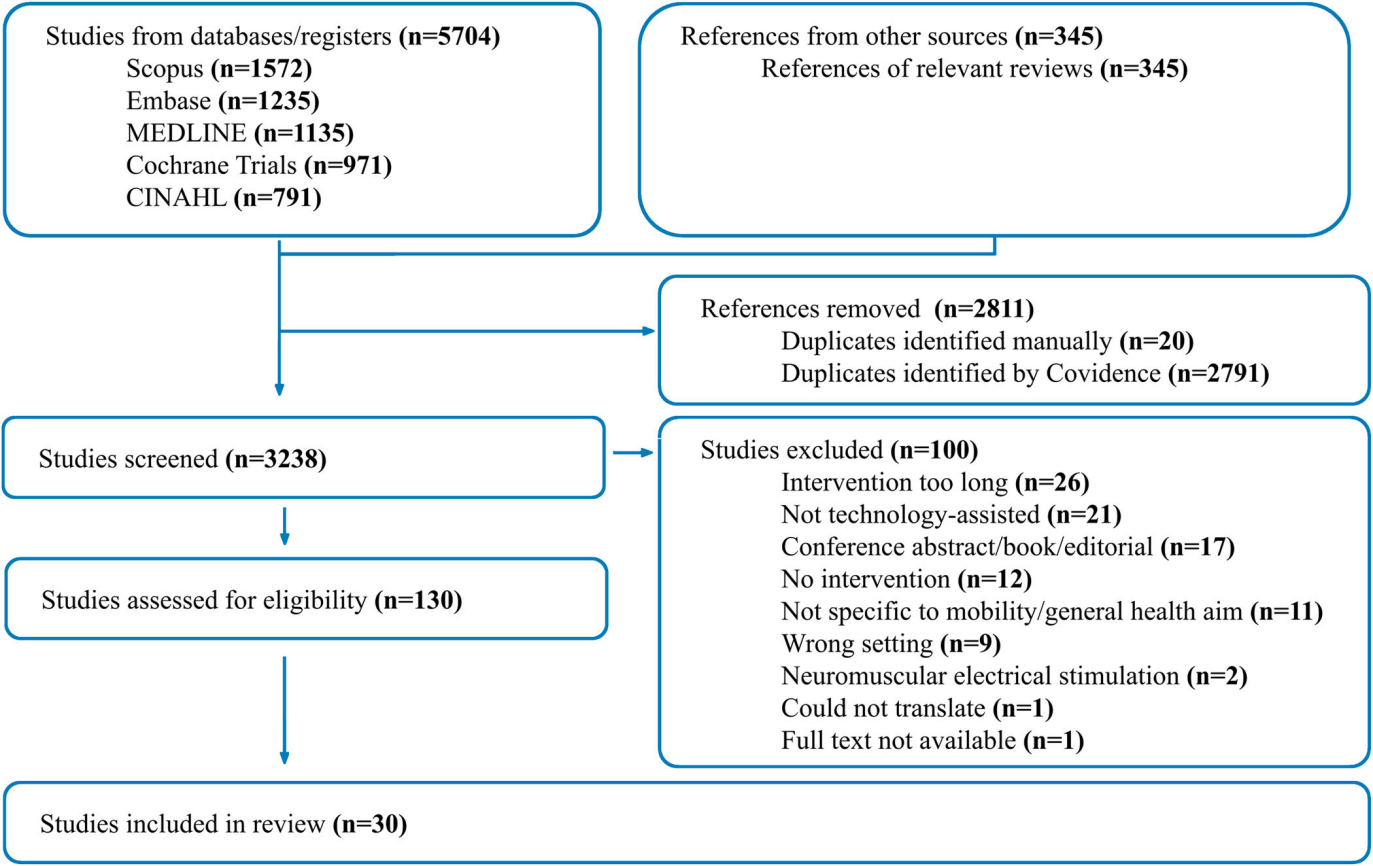
Preferred Reporting Items for Systematic Reviews and Meta‐Analysis (PRISMA) Diagram. Originally created by Covidence and edited to account for the inclusion of results from citation chasing of reviews.

A health sciences librarian (L. D.) conducted searches of Medline, EMBASE, CINAHL, Scopus, and the Cochrane trials database on June 26, 2024, for three search concepts: technology‐assisted, mobility/ambulation, and inpatient (File S1, Section 3). All citations were exported into Covidence (a web‐based systematic review platform for importing and independent screening of papers), and duplicates were removed. Two reviewers (P. M. and A. W.) screened 10 articles together to ensure consistency, and then title and abstract screening were independently conducted. Citation tracking using Scopus and citationchaser was performed on 18 relevant reviews found during the initial screening, and the results (*n* = 345) of this were screened using the same method. Disagreements during the screening process were resolved through discussion. The results selected for full‐text review were reviewed using the same process. The preferred reporting items for systematic reviews and meta‐analyses extension for scoping reviews (PRISMA‐ScR flow diagram is detailed in Figure 1).

### Data extraction and analysis

Two reviewers (P. M. and A. W.) developed a data extraction form a priori and to evaluate the tool, five included studies were selected for variability, resulting in the finalized tool (File S1, Section 3). Two authors (A. W. and P. M.) extracted three articles independently and discussed disagreements in an initial piloting stage. Extraction of behavior change techniques and implementation strategies was completed independently by two authors (A. W. and P. M.), though extraction of other variables was completed by A. W. and reviewed by P. M. due to time constraints. Disagreements were resolved through consensus. Another team member (N. K.) confirmed relevant papers and data extraction. The variables collected included the first author's name, year of publication, article title, country in which the intervention occurred, type of hospital inpatient setting, patient demographics, intervention characteristics (e.g., summary, start time, clinicians involved, effectiveness outcomes), and implementation factors (e.g., framework used, strategies, and implementation outcomes). Implementation outcomes were defined according to Proctor's taxonomy.^21^ A descriptive analysis was conducted using Microsoft Excel (version 2021) summarizing the data. Descriptions of interventions were inductively coded for behavior change techniques, which refer to patient‐level implementation. Extracted implementation strategies, which refer to facility‐level implementation, were deductively coded according to the Expert Recommendations for Implementing Change.^22^ A study flow diagram can be found in File S1, Section 1.

## RESULTS

From 5704 citation results, 30 articles were included in this review. Of these, two pairs of articles^11^, ^12^, ^23^, ^24^ represent the same initiative; therefore, 28 unique initiatives were identified (Figure 1). Nine trial registrations, three published protocols, and 18 published articles were included. Of the published articles, 59% (*n* = 10) were randomized controlled trials (RCTs), and all published protocols were for RCTs. Included articles represent 11 countries distributed across four world regions: Europe (*n* = 13), North America (*n* = 8), Asia (*n* = 7), and Australia (*n* = 2). All North American results were from the United States of America. Patient populations varied widely with the most common specified category being postoperative (*n* = 13), followed by stroke (*n* = 5). The most common age range was 60–70 years (*n* = 9), and no studies had an average age under 40 (Table 1). The number of trial registrations and publications about technology‐assisted interventions has increased over time (File S1, Section 5), indicating that this is an emerging field. The earliest identified article was published in 2015, with the highest number of studies published in 2023 (*n* = 6).

**Table 1:** Characteristics of included studies.

Author and year OR trial ID and year	Country	Study design	Patient population	Mean age a	Technology used b	Intervention start
ACTRN12619001151123 2019^25^	Australia	TR; RCT	Cancer	NR	STEP	NR
Conijn et al., 2020^10^	Netherlands	QuasiExperimental Pilot	Transplantation and vascular surgery	58	STEP	Day 1 post‐op
Cuevas‐Lara et al., 2022^7^	Spain	Non‐RCT (open label)	Pulmonary cardiology and neurology	86	GAME	NR
Dall et al., 2019^26^	Denmark	Randomized Study	COPD and pneumonia	73	STEP	Admission day
Dong et al., 2018^27^	China	Protocol; RCT	Stroke	NR	STEP	1 day after admission
DRKS00032238 2023^28^	Germany	TR; RCT	Stroke	NR	STEP	NR
Fan, et al., 2023^8^	Singapore	RCT	Medicine	77	GAME	NR
Ghio et al., 2021^13^	USA	RCT	Surgery	42	TELE	Day 2 post‐op
ISRCTN 96991582 2015^29^	USA	TR; RCT	Bariatric surgery	NR	STEP	NR
Kanai et al., 2017^11^	Japan	Pilot Study	Stroke	63	STEP	NR
Kanai et al., 2018^12^	Japan	RCT	Stroke	65	STEP	Mean of 3.7 days after admission
Karlsson et al., 2023^30^	Sweden	Non‐RCT	Abdominal cancer surgery	69	APP	NR
NCT02833324 2016^31^	USA	TR	Colorectal surgery	NR	STEP, TELE	Same day as surgery
NCT03595605 2018^32^	USA	TR	Medicine	NR	STEP, TELE	NR
NCT04444453 2020^33^	USA	TR	Medicine	NR	STEP	Day 1 after admission
NCT04555330 2020^34^	Denmark	TR	Pulmonary medicine, cardiology and geriatrics	NR	STEP	NR
NCT04743401 2021^35^	USA	TR	COVID‐19	NR	GAME	NR
NCT06407427 2024^36^	USA	TR	Orthopedic surgery	NR	APP	Day 1 post‐op
No et al., 2021^37^	Korea	RCT	Gynecology surgery	54	STEP	2 days pre‐op
Reed et al., 2021^38^	Japan	RCT	Bariatric surgery	44	STEP	4‐6 h post‐op
Schrempf et al., 2023^9^	Germany	RCT	Colorectal cancer surgery	61	GAME	Day 1 post‐op
Schwab et al., 2020^39^	Germany	Protocol; RCT	Abdominal surgery	NR	STEP	NR
Takei et al., 2024^40^	Japan	Repeated measure mixed methods	Geriatric	79	GAME	NR
Thomas & Wolfe 2017^41^	USA	Preliminary study	Heart disease and stroke	68	GAME	NR
Van der Walt et al, 2018^42^	Australia	RCT	Hip and knee surgery	66	STEP	2 weeks pre‐op
Van Dijk‐Huisman et al., 2020^23^	Netherlands	RCT	Orthopedic surgery	66	STEP, APP	Before or on the day of surgery
Van Dijk‐Huisman et al., 2023^24^	Netherlands	RCT	Pulmonology and internal medicine	63	STEP, APP	NR
Van Grootel et al., 2023^43^	Netherlands	Pre‐post study design	Pulmonology and nephrology	60	STEP	Within two days of admission
Wiklund et al., 2015^44^	Sweden	RCT	Gastric bypass surgery	42	STEP	Day 1 post‐op
Wolf et al., 2022^45^	Germany	Protocol; RCT	Colorectal and liver cancer surgery	NR	GAME	Day 1 post‐op

Abbreviations: NR, not reported; PT, physical therapist; RCT, randomized controlled trials; Tech, technology; TR, trial registration.

^a^ Mean age was rounded to the nearest year.

^b^ Technology coding: APP, application for in‐bed conditioning exercises; GAME, virtual reality and/or exergames (e.g., sit‐to‐stand games, Health Arcade, rowing simulation); TELE, mobile or telephone ambulation reminders; STEP, step or activity tracking device.

Four main technologies: step count/activity trackers,^11^, ^12^, ^23^, ^24^, ^25^, ^26^, ^27^, ^28^, ^29^, ^31^, ^32^, ^33^, ^34^, ^37^, ^38^, ^39^, ^42^, ^43^, ^44^ mobile/telephone ambulation reminders,^13^, ^31^, ^32^ exergames,^7^, ^8^, ^35^, ^40^, ^41^, ^45^ or apps for in‐bed exercises^23^, ^24^, ^30^, ^36^ were identified. Fitbits^10^, ^11^, ^12^, ^38^ and ActivePal^25^, ^30^ devices, and the MOX activity monitor^23^, ^24^ were the most frequently used (File S1, Section 6). Interventions using activity trackers often included a goal‐setting component, with goals either standardized for all patients,^11^, ^12^, ^32^ on baseline physical activity,^37^ or determined through consultation with a health care professional.^28^ In addition, all but one study^33^ using an activity tracker allowed the patient to receive feedback on how much they had moved. Some ambulation prompts were simple reminders to ambulate such as “please take more steps than yesterday”^39^ while others included patient education components, such as “walking is great medicine!”^13^ Several types of exergames were used, ranging from color matching^8^ to hunting games.^41^ Two studies^9^, ^45^ used virtual reality technology as part of the exergame intervention. Two studies solely used exercise apps,^30^, ^36^, while an additional two combined them with activity trackers^23^, ^24^ (Table 1).

Many articles did not specify when the interventions began relative to patient hospital admission (*n* = 14). The most common timeframe for intervention start was the day after admission or surgery (*n* = 7); some interventions started the day of (*n* = 4), later in the hospital stay (*n* = 3), or approached patients before their surgeries for consent (*n *= 2) (Table 1).

The most reported technique (*n* = 8) involved the support of a physical therapist, who facilitated collaborative goal setting,^28^ encouraging and educating patients,^26^ and monitoring and adapting the intervention.^10^, ^11^, ^12^, ^23^, ^24^ Collaborative goal setting (*n* = 3) involved the patient and a health care provider deciding together on realistic goals that are personally motivating for the patient. Patient education (*n* = 5) often involved topics such as the importance of ambulation^11^, ^25^, ^28^, ^31^ and could be completed through a discussion with a health care provider^10^, ^28^ or through the provision of educational materials such as brochures^10^, ^25^, ^28^ or digital resources.^10^

The second most common technique was increasing daily goals (*n* = 6), which were primarily used in stroke^11^, ^12^, ^27^ or postsurgical settings.^37^, ^44^ Seven studies^11^, ^12^, ^23^, ^24^, ^30^, ^38^, ^44^ used progress tracking to motivate patients, where participants were provided with or created a record displaying how much they had increased their physical activity since admission. Of those, three studies^11^, ^12^, ^44^ combined progress tracking with increasing activity goals. Feedback to the patients on their movement levels was also shown to visitors, other patients, and staff using a visual data display (*n* = 5) on a bedside tablet^26^, ^30^, ^34^ or on a TV located on the ward (Table 2). ^28^, ^43^

**Table 2:** Approaches to enable physical activity behavior change.

Author and year OR trial ID and year	Encouragement	Collaborative goal setting	Increasing daily goals	Progress tracking	Visual data display	Environmental modification	PT supported	Patient education	Total
ACTRN12619001151123 2019^25^		✓					✓	✓	3
Conijn et al., 2020^10^					✓		✓	✓	3
Cuevas‐Lara et al., 2022^7^									NR
Dall et al., 2019^26^					✓		✓		2
Dong et al., 2018^27^			✓						1
DRKS00032238 2023^28^		✓			✓		✓	✓	4
Fan, et al., 2023^8^									NR
Ghio et al., 2021^13^								✓	1
ISRCTN96991582 2015^29^									NR
Kanai et al 2017^11^	✓	✓	✓	✓			✓		5
Kanai et al., 2018^12^	✓	✓	✓	✓			✓		5
Karlsson et al., 2023^30^				✓					1
NCT02833324 2016^31^								✓	1
NCT03595605 2018^32^									NR
NCT04444453 2020^33^									NR
NCT04555330 2020^34^					✓				1
NCT04743401 2021^35^									NR
NCT06407427 2024^36^									NR
No et al., 2021^37^			✓						1
Reed et al., 2021^38^				✓					1
Schrempf et al., 2023^9^									NR
Schwab et al., 2020^39^									NR
Takei et al., 2024^40^									NR
Thomas et al., 2017^41^									NR
Van der Walt et al, 2018^42^									NR
van Dijk‐Huisman et al., 2020^23^				✓			✓		2
van Dijk‐Huisman et al., 2023^24^				✓			✓		2
van Grootel et al., 2023^43^			✓		✓	✓			3
Wiklund et al., 2015^44^			✓	✓					2
Wolf et al., 2022^45^									NR
Total	2	4	6	7	5	1	8	5	38

Abbreviation: NR, not reported.

Lastly, two studies^11^, ^12^ explicitly employed encouragement from staff as a strategy, while one study^43^ employed environmental modification in the form of marking walking routes around the ward and putting up posters with recommended exercises (File S1, Section 7).

Physical activity outcomes were measured through steps per day (*n* = 12), time spent out of bed (*n* = 11), patient compliance to exercise programs (*n* = 4), and time to first ambulation (*n* = 2). Nine studies measured some variation of physical function, while four measured quality of life. Included articles also measured length of stay (*n *= 13), incidence of complications (*n* = 9), patient satisfaction (*n* = 5), and readmission rates (*n* = 4). For a full list of all outcomes measured by included studies, please refer to File S2. Six studies^9^, ^10^, ^30^, ^40^, ^43^, ^45^ reported implementation outcomes, with most reporting two or more.^9^, ^10^, ^30^, ^43^ In order of frequency of use, those outcomes were: feasibility (*n* = 4), acceptability (*n* = 3), adoption (*n* = 2), and penetration (*n* = 1). The remaining outcomes identified in Proctor's taxonomy^21^ (appropriateness, cost, fidelity, and sustainability) were not reported by any included study.

Additionally, some studies reported implementation strategies,^10^, ^30^, ^43^ which are designed to support implementing the intervention into clinical practice.^15^ Five strategies from the Expert Recommendations for Implementing Change^22^ were identified in this review, with each study reporting between one and five strategies (Table 3). The most common strategy, conducting educational meetings, involved training ward staff in intervention procedures. The second most common strategy involved the distribution of educational materials. This included distributing protocols for the staff working closely with the intervention to use, as well as more general materials for staff who would be nearby but not directly involved. In addition, the study reporting the highest number of implementation strategies^43^ also considered the following strategies: identifying and preparing a champion, facilitating relay of clinical data to providers, and developing educational materials. This “champion” was a designated, experienced nurse who was responsible for building support on the unit, answering questions about the intervention, and incorporating the intervention into standard care. To facilitate the relay of data, physical activity data were incorporated into regular team meetings. Furthermore, educational materials were created for the health care providers who would be administering the intervention (File S1, Section 8).

**Table 3:** Expert recommendations for implementing change strategies identified per article.

Author and Year OR Trial ID and Year	Identify and prepare clinical champions	Facilitate relay of clinical data to providers	Conduct educational meetings	Develop educational materials	Distribute Educational Materials	Total
Conijn et al., 2020^20^			✓		✓	2
Van Grootel et al., 202^43^	✓	✓	✓	✓	✓	5
Karlsson et al., 2023^30^			✓			1
Total	1	1	3	1	2	8

Three studies used theory to inform the selection of either behavior change techniques or implementation strategies. One study^43^ used intervention mapping, an approach guiding the creation, implementation, and assessment of an intervention.^46^ Additionally, two studies used self‐efficacy theory to guide their selection of behavior change techniques.^11^, ^12^

## DISCUSSION

Technology‐assisted mobility interventions are an emerging area of research in the hospital setting; studies meeting inclusion criteria were all published in the last decade, and several were protocols or trial registrations. We identified four distinct technology‐assisted mobility interventions (activity trackers, ambulation prompts, exergames, and applications for in‐bed exercises). Additionally, eight patient behavior change techniques (encouragement, collaborative goal setting, increasing daily goals, progress tracking, visual data displays, physiotherapist support, and patient education) were identified. Only two studies used theory to inform or justify behavior change techniques, despite evidence suggesting theory‐informed interventions are more effective,^47^, ^48^ because of the alignment of a specific behavioral determinant with appropriate strategies. For example, if patients′ sedentary behavior stems from a fear of falling, identifying this barrier could prompt environmental modifications such as installing grab bars and placing chairs along walking routes.

Five implementation strategies (identifying and preparing clinical champions, facilitating the relay of clinical data, conducting educational meetings, and creating and distributing educational materials) were reported by included studies. This summary may help clinicians and researchers identify and select technology‐assisted interventions and strategies that best align with their patient population, clinical setting, and available resources. Notably, few implementation strategies were reported, with the majority of studies not reporting the implementation approach taken. To enable comparison of implementation plans and strategies, future studies should provide more detailed reporting. Adherence to established reporting guidelines will facilitate both meta‐analyses and replication, thereby advancing implementation research.^15^ Additionally, most of the studies which did report implementation strategies did not use theories or frameworks to support or inform initiative development.

This study presents a list of implementation strategies that have been used to support technology‐assisted mobility interventions; however, it is not intended to be an exhaustive list, especially given that few included studies explicitly addressed implementation. Identifying barriers and facilitators in the local context and considering implementation strategies in advance may optimize the success of implementation. Implementation theories may also help support a systematic decision‐making process for choosing which strategies are best suited for a given context.^49^

There were limitations to synthesizing findings across the articles. The use of technology‐assisted interventions is still relatively new in healthcare, and the variability in reporting made comparisons challenging. Our strict inclusion criteria, focused on specific healthcare settings, may have resulted in the exclusion of relevant studies. While the inclusion of trial registrations helped highlight the field's ongoing development, these often lacked the detail found in full published protocols, making it difficult to fully describe the interventions. Unfortunately, due to time constraints, we were unable to reach out to the authors of the included protocol studies for clarification on implementation strategies or behavior change techniques that may have been used but not reported. While our findings offer valuable insights, the use of a behavior change framework could have further enhanced the study by providing a structured theoretical lens through which to categorize and interpret the identified behavior change techniques.

## CONCLUSIONS

The implementation of technology‐assisted mobility interventions in hospital settings to enhance patient mobility is an emerging area of research. As such, there is currently limited evidence to definitively determine feasibility. However, there is considerable opportunity to improve the use of theoretical frameworks to inform the selection of both behavior change techniques and implementation strategies, which may improve the effectiveness of these initiatives. Additionally, consistent and detailed reporting of implementation strategies would support replication and local adaptation of initiatives. Future studies are required to evaluate implementation strategy outcomes and to examine patient and clinician experiences to inform intervention adaptation and to facilitate integration into routine clinical hospital ward/unit practice.

## CONFLICT OF INTEREST STATEMENT

The authors declare no conflicts of interest.

## ETHICS STATEMENT

Ethical approval was not applicable.

## Supporting information

Mobility Scoping Review ‐ Supplemental File 1 (Revised) (1).docx.

Supplemental File 1 ‐ Figure for Section 1.tiff.

Supplemental File 1‐ Figure for Section 5.tiff.

Supplemental File 2 (Nov 8).xlsx.
