# Supplementary material for: Implementing in hospital technology‐assisted mobility initiatives: A scoping review
Source: J Hosp Med. 2026 Jan 25;21(4):413–22. doi: 10.1002/jhm.70262 (PMC13064589; doi:10.1002/jhm.70262)
Supplement: Supplementary file 1 — Mobility Scoping Review ‐ Supplemental File 1 (Revised) (1).docx. [file JHM-21-413-s003.docx]

**In Hospital Technology-Assisted Mobility Initiatives: A Scoping Review**

**Supplemental File 1**

Section 1. Overview of study process


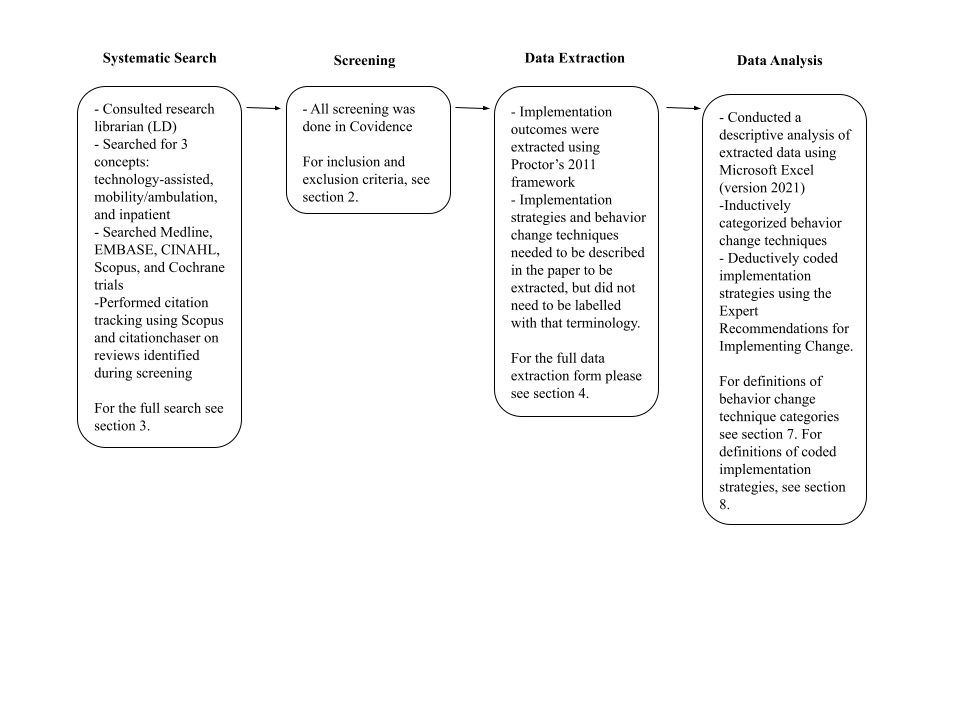


Section 2. Inclusion and exclusion criteria

| Inclusion | Exclusion |
| --- | --- |
| 1. Describes a technology-assisted intervention aimed at increasing mobility (physical activity) or decreasing sedentary behavior. 2. Intervention takes place in hospital care setting (adult inpatients). 3. Protocols which meet criteria but whose full-length studies are not yet published/not available. 4. Peer reviewed articles, all study designs (i.e., qualitative, quantitative, and mixed methods) | 1. Reviews of any kind  2. Setting is over 50% pediatric.  3. Protocols of full studies also included in the review.  4. Conference proceedings, books, letters to the editor.  5.Intervention in non-hospital settings (i.e., clinic), rehabilitation hospital settings, ICU’s or maternity wards.  6. Intervention is longer than 14 days.  7.Mobility educational interventions  9. Studies examining prehabilitation or pre-operative programs.  10. Article is not available in English and does not translate well with Google Translate. |

Section 3. Full Search Strategy

| **Database** | **Search Strategy** |
| --- | --- |
| **Medline**  June 26, 2024 | 1 Inpatients/ 31269  2 Hospitalization/ 141095  3 hospital units/ or burn units/ or coronary care units/ or recovery room/ or respiratory care units/ or hospitals/ or hospitals, community/ or hospitals, general/ or hospitals, high-volume/ or hospitals, low-volume/ or exp hospitals, public/ or hospitals, special/ or cancer care facilities/ or cardiac care facilities/ or hospitals, teaching/ or hospitals, university/ or hospitals, urban/ or tertiary care centers/ 281904  4 (inpatient* or hospitalized or hospitalised or "hospital stay" or "in hospital" or (hospital* adj3 (patient* or adult*)) or ((step-down or stepdown or step-up or "high dependency" or telemetry or intermediate or "transitional care") adj2 (unit or units or ward or wards or room or rooms)) or ((hospital or medical or burn or cardiac or surgical or surgery or neuro* or cancer or endocrinology or dialysis or hematolog* or haematolog*) adj2 (unit or units or ward or wards))).mp. 627715  5 (admitted adj3 (patient or patients)).mp. 114422  6 1 or 2 or 3 or 4 or 5 973901  7 ((pediatric or paediatric or child* or teen* or adolescen* or infant* or neonat*) not adult*).ti,bt,kf. 1671898  8 limit 7 to "all child (0 to 18 years)" 1359380  9 limit 8 to "all adult (19 plus years)" 166010  10 6 not ((8 not 9) or 7) 886950  11 (pregnan* or preoperative or pre-operative or prehabilitation).ti,bt,kf. 389240  12 10 not 11 875182  13 Early Ambulation/ 3362  14 Sedentary Behavior/ 14302  15 movement/ or locomotion/ or motor activity/ or exercise/ or muscle stretching exercises/ or walking/ or physical exertion/ 438186  16 ("early ambulation" or "early mobilization" or "early mobilisation" or "early progressive mobilisation" or "early progressive mobilization" or ((reduc* or prevent* or decreas* or barriers or facilitators or intervention* or "quality improvement" or strategy or strategies or program* or initiative* or approach or approaches or model or models or implement*) adj5 (immobile or immobility or sedentary)) or ((barriers or facilitators or increase or improv* or enabl* or determin* or evaluat* or assess* or track* or monitor* or intervention* or "quality improvement" or strategy or strategies or program* or initiative* or approach or approaches or model or models or implement*) adj5 (mobility or movement or mobilization or mobilisation)) or (exercise* not ("breathing exercise*" or "visual* exercise*" or "writing exercise*")) or "physical* activ*" or (activity adj3 level*) or "motor activity" or walking or "daily step*" or "steps per day" or "step count" or "step counts" or "step volume" or ((bed or bedside) adj3 cycle*) or cycling or bicycl* or exergam* or ambulation or ambulating or ambulate* or locomotion).mp. 1019597  17 13 or 14 or 15 or 16 1109618  18 wearable electronic devices/ or fitness trackers/ 10124  19 (pedomet* or acceleromet* or actigraph* or activpal* or fitbit* or "activity-tracker*" or "fitness tracker*" or step-counter* or wearable* or "activity monitor*" or "fitness monitor*" or tech*-enabled or tech*-based or tech*-supported or tech*-assisted or (technolog* adj3 (intervention* or implement* or innovative or emerging or digital or portable)) or app or apps or (application* adj3 (phone* or mobile or digital or online or web or electronic)) or smartphone* or "smart phone*" or "smart watch*" or smartwatch* or ((wrist* or wear or worn) adj3 (device* or monitor* or sensor*)) or mobile-device* or "cell* phone" or "mobile phone*" or ipad* or iphone* or tablet or "electronic device*" or "handheld device*" or "hand-held device*" or gps or "global positioning system*" or sms or "short messag* service" or texting or texts or texted or "text messag*" or gamification or exergam*).mp. 360178  20 18 or 19 360178  21 12 and 17 and 20 1198  22 21 not ((home-based or outpatient* or tele or telephone or telecare or telehealth or telemedicine or telerehab* or teleexercis* or telemonitor*) not (inpatient* or hospital* or ward or wards)).ti,bt,kf. 1135 |
| **EMBASE**  June 26, 2024 | 1 hospital patient/ or aged hospital patient/ 241637  2 ward/ or geriatric ward/ or high dependency unit/ or intermediate care unit/ or observation unit/ or oncology ward/ 27938  3 *hospital/ or *burn care hospital/ or *community hospital/ or *general hospital/ or *geriatric hospital/ or *high volume hospital/ or *non profit hospital/ or *private hospital/ or *public hospital/ or *rural hospital/ or exp *teaching hospital/ or *urban hospital/ 141347  4 (inpatient* or hospitalized or hospitalised or "hospital stay" or "in hospital" or (hospital* adj3 (patient* or adult*)) or ((step-down or stepdown or step-up or "high dependency" or telemetry or intermediate or "transitional care") adj2 (unit or units or ward or wards or room or rooms)) or ((hospital or medical or burn or cardiac or surgical or surgery or neuro* or cancer or endocrinology or dialysis or hematolog* or haematolog*) adj2 (unit or units or ward or wards))).tw,kf. 1013788  5 (admitted adj3 (patient or patients)).mp. 217016  6 1 or 2 or 3 or 4 or 5 1316148  7 ((pediatric or paediatric or child* or teen* or adolescen* or infant* or neonat*) not adult*).ti,bt,kf. 1968290  8 limit 6 to child <unspecified age> 124947  9 limit 8 to (adult <18 to 64 years> or aged <65+ years>) 46775  10 (6 not (8 not 9)) or (6 not 7) 1258435  11 (pregnan* or preoperative or pre-operative or prehabilitation).ti,bt,kf. 458429  12 10 not 11 1240165  13 mobilization/ 41990  14 body movement/ 15843  15 locomotion/ or walking/ 175120  16 motor activity/ 48678  17 exercise/ or aerobic exercise/ or arm exercise/ or leg exercise/ or low intensity exercise/ or moderate intensity exercise/ or exp muscle exercise/ 397952  18 ("early ambulation" or "early mobilization" or "early mobilisation" or "early progressive mobilisation" or "early progressive mobilization" or ((reduc* or prevent* or decreas* or barriers or facilitators or intervention* or "quality improvement" or strategy or strategies or program* or initiative* or approach or approaches or model or models or implement*) adj5 (immobile or immobility or sedentary)) or ((barriers or facilitators or increase or improv* or enabl* or determin* or evaluat* or assess* or track* or monitor* or intervention* or "quality improvement" or strategy or strategies or program* or initiative* or approach or approaches or model or models or implement*) adj5 (mobility or movement or mobilization or mobilisation)) or (exercise* not ("breathing exercise*" or "visual* exercise*" or "writing exercise*")) or "physical* activ*" or (activity adj3 level*) or "motor activity" or walking or "daily step*" or "steps per day" or "step count" or "step counts" or "step volume" or ((bed or bedside) adj3 cycle*) or cycling or bicycl* or exergam* or ambulation or ambulating or ambulate* or locomotion).mp. 1436726  19 13 or 14 or 15 or 16 or 17 or 18 1467551  20 wearable device/ or exp wearable computer/ or wearable sensor/ 16152  21 accelerometer/ 19562  22 exp activity tracker/ 5470  23 (pedomet* or acceleromet* or actigraph* or activpal* or fitbit* or "activity-tracker*" or "fitness tracker*" or step-counter* or wearable* or "activity monitor*" or "fitness monitor*" or tech*-enabled or tech*-based or tech*-supported or tech*-assisted or (technolog* adj3 (intervention* or implement* or innovative or emerging or digital or portable)) or app or apps or (application* adj3 (phone* or mobile or digital or online or web or electronic)) or smartphone* or "smart phone*" or "smart watch*" or smartwatch* or ((wrist* or wear or worn) adj3 (device* or monitor* or sensor*)) or mobile-device* or "cell* phone" or "mobile phone*" or ipad* or iphone* or tablet or "electronic device*" or "handheld device*" or "hand-held device*" or gps or "global positioning system*" or sms or "short messag* service" or texting or texts or texted or "text messag*" or gamification or exergam*).mp. 513630  24 20 or 21 or 22 or 23 515213  25 12 and 19 and 24 2276  26 25 not ((home-based or outpatient* or tele or telephone or telecare or telehealth or telemedicine or telerehab* or teleexercis* or telemonitor*) not (inpatient* or hospital* or ward or wards)).ti,bt,kf. 2142  27 limit 26 to conference abstracts 907  28 26 not 27 1235 |
| **CINAHL** Plus with Full Text (EBSCO Host)  June 26, 2024 | S1 (MH "Inpatients") OR (MH "Burn Units") OR (MH "Hospital Units") OR (MH "Observation Units") OR (MH "Oncology Care Units") or (MH "Hospitals") OR (MH "Hospitals, Community") OR (MH "Hospitals, Private") OR (MH "Hospitals, Public") OR (MH "Hospitals, Veterans") OR (MH "Hospitals, Rural") OR (MH "Cancer Care Facilities") OR (MH "Hospitals, Urban")  S2 inpatient* or hospitalized or hospitalised or "hospital stay" or "in hospital" or (hospital* N3 (patient* or adult*)) or ((step-down or stepdown or step-up or "high dependency" or telemetry or intermediate or "transitional care") N2 (unit or units or ward or wards or room or rooms)) or ((hospital or medical or burn or cardiac or surgical or surgery or neuro* or cancer or endocrinology or dialysis or hematolog* or haematolog*) N2 (unit or units or ward or wards)) or (admitted N3 (patient or patients))  S3 S1 OR S2  S4 ("early ambulation" or "early mobilization" or "early mobilisation" or "early progressive mobilisation" or "early progressive mobilization" or ((reduc* or prevent* or decreas* or barriers or facilitators or intervention* or "quality improvement" or strategy or strategies or program* or initiative* or approach or approaches or model or models or implement*) N5 (immobile or immobility or sedentary)) or ((barriers or facilitators or increase or improv* or enabl* or determin* or evaluat* or assess* or track* or monitor* or intervention* or "quality improvement" or strategy or strategies or program* or initiative* or approach or approaches or model or models or implement*) N5 (mobility or movement or mobilization or mobilisation)) or (exercise* not ("breathing exercise*" or "visual* exercise*" or "writing exercise*")) or "physical* activ*" or (activity N3 level*) or "motor activity" or walking or "daily step*" or "steps per day" or "step count" or "step counts" or "step volume" or ((bed or bedside) N3 cycle*) or cycling or bicycl* or exergam* or ambulation or ambulating or ambulate* or locomotion)  S5 ( (MH "Fitness Trackers") OR (MH "Wearable Sensors") OR (MH "Accelerometers") ) OR ( pedomet* or acceleromet* or actigraph* or activpal* or fitbit* or "activity-tracker*" or "fitness tracker*" or step-counter* or wearable* or "activity monitor*" or "fitness monitor*" or tech*-enabled or tech*-based or tech*-supported or tech*-assisted or (technolog* N3 (intervention* or implement* or innovative or emerging or digital or portable)) or app or apps or (application* N3 (phone* or mobile or digital or online or web or electronic)) or smartphone* or "smart phone*" or "smart watch*" or smartwatch* or ((wrist* or wear or worn) N3 (device* or monitor* or sensor*)) or mobile-device* or "cell* phone" or "mobile phone*" or ipad* or iphone* or tablet or "electronic device*" or "handheld device*" or "hand-held device*" or gps or "global positioning system*" or sms or "short messag* service" or texting or texts or texted or "text messag*" or gamification or exergam*  S6 S3 AND S4 AND S5  S7 ( ((home-based or outpatient* or tele or telephone or telecare or telehealth or telemedicine or telerehab* or teleexercis* or telemonitor*) not (inpatient* or hospital* or ward or wards)) ) OR ( TI(pregnan* or preoperative or pre-operative or prehabilitation) ) OR ( TI((pediatric or paediatric or child* or teen* or adolescen* or infant* or neonat*) not adult*) ) OR ( ((MH "Child+") OR (MH "Adolescence+")) NOT (MH "Adult+") )  S8 S6 NOT S7 |
| **Cochrane Library**, (Trials Database)  June 26, 2024 | #1 [mh ^"Inpatients"] or [mh ^"hospital units"] or [mh ^"burn units"] or [mh ^"coronary care units"] or [mh ^"recovery room"] or [mh ^"respiratory care units"] or [mh ^"hospitals"] or [mh ^"hospitals, community"] or [mh ^"hospitals, general"] or [mh ^"hospitals, high-volume"] or [mh ^"hospitals, low-volume"] or [mh "hospitals, public"] or [mh ^"hospitals, special"] or [mh ^"cancer care facilities"] or [mh ^"cardiac care facilities"] or [mh ^"hospitals, teaching"] or [mh ^"hospitals, university"] or [mh ^"hospitals, urban"] or [mh ^"tertiary care centers"]  #2 (inpatient* or hospitalized or hospitalised or "hospital stay" or "in hospital" or (hospital* NEAR/3 (patient* or adult*)) or ((step-down or stepdown or step-up or "high dependency" or telemetry or intermediate or "transitional care") NEAR/2 (unit or units or ward or wards or room or rooms)) or ((hospital or medical or burn or cardiac or surgical or surgery or neuro* or cancer or endocrinology or dialysis or hematolog* or haematolog*) NEAR/2 (unit or units or ward or wards))):ti,ab,kw  #3 (admitted NEAR/3 (patient or patients)):ti,ab,kw  #4 #1 OR #2 OR #3  #5 #4 NOT ([mh "child"] NOT [mh "adult"])  #6 [mh ^"Early Ambulation"] OR [mh ^"Sedentary Behavior"] OR [mh ^"movement"] or [mh ^"locomotion"] or [mh ^"motor activity"] or [mh ^"exercise"] or [mh ^"muscle stretching exercises"] or [mh ^"walking"] or [mh ^"physical exertion"] OR ("early ambulation" or "early mobilization" or "early mobilisation" or "early progressive mobilisation" or "early progressive mobilization" or ((reduc* or prevent* or decreas* or barriers or facilitators or intervention* or "quality improvement" or strategy or strategies or program* or initiative* or approach or approaches or model or models or implement*) NEAR/5 (immobile or immobility or sedentary)) or ((barriers or facilitators or increase or improv* or enabl* or determin* or evaluat* or assess* or track* or monitor* or intervention* or "quality improvement" or strategy or strategies or program* or initiative* or approach or approaches or model or models or implement*) NEAR/5 (mobility or movement or mobilization or mobilisation)) or (exercise* not ((breathing NEXT exercise*) or (visual* NEXT exercise*) or (writing NEXT exercise*))) or (physical* NEXT activ*) or (activity NEAR/3 level*) or "motor activity" or walking or (daily NEXT step*) or "steps per day" or "step count" or "step counts" or "step volume" or ((bed or bedside) NEAR/3 cycle*) or cycling or bicycl* or exergam* or ambulation or ambulating or ambulate* or locomotion):ti,ab,kw  #7 [mh ^"wearable electronic devices"] or [mh ^"fitness trackers"] OR (pedomet* or acceleromet* or actigraph* or activpal* or fitbit* or (activity NEXT tracker*) or (fitness NEXT tracker*) or step-counter* or wearable* or (activity NEXT monitor*) or (fitness NEXT monitor*) or (tech* NEXT (enabled or based or supported or assisted)) or (technolog* NEAR/3 (intervention* or implement* or innovative or emerging or digital or portable)) or app or apps or (application* NEAR/3 (phone* or mobile or digital or online or web or electronic)) or smartphone* or (smart NEXT phone*) or (smart NEXT watch*) or smartwatch* or ((wrist* or wear or worn) NEAR/3 (device* or monitor* or sensor*)) or (mobile NEXT device*) or (cell* NEXT phone*) or (mobile NEXT phone*) or ipad* or iphone* or tablet or (electronic NEXT device*) or (handheld NEXT device*) or (hand-held NEXT device*) or gps or ("global positioning" NEXT system*) or sms or "short message service" or texting or texts or texted or (text NEXT messag*) or gamification or exergam*):ti,ab,kw  #8 #5 AND #6 AND #7  #9 ((home-based or outpatient* or tele or telephone or telecare or telehealth or telemedicine or telerehab* or teleexercis* or telemonitor*) not (inpatient* or hospital* or ward or wards)):ti  #10 ((pediatric or paediatric or child* or teen* or adolescen* or infant* or neonat*) not adult*):ti  #11 (pregnan* or preoperative or presurgical or pre-surgical or pre-operative or prehabilitation):ti  #12 #8 NOT (#9 OR #10 OR #11) |
| **Scopus,**  June 26, 2024 | ( KEY ( inpatient* OR "hospital patient" OR ward ) OR TITLE-ABS ( inpatient* OR hospitalized OR hospitalised OR "hospital stay" OR {in hospital} OR ( hospital* W/3 ( patient* OR adult* ) ) OR ( ( step-down OR stepdown OR step-up OR "high dependency" OR telemetry OR intermediate OR "transitional care" ) W/2 ( unit OR units OR ward OR wards OR room OR rooms ) ) OR ( ( hospital OR medical OR burn OR cardiac OR surgical OR surgery OR neuro* OR cancer OR endocrinology OR dialysis OR hematolog* OR haematolog* ) W/2 ( unit OR units OR ward OR wards ) ) OR ( admitted W/3 ( patient OR patients ) ) ) ) AND TITLE-ABS ( "early ambulation" OR "early mobilization" OR "early mobilisation" OR "early progressive mobilization" OR "early progressive mobilisation" OR ( ( reduc* OR prevent* OR decreas* OR barriers OR facilitators OR intervention* OR "quality improvement" OR strategy OR strategies OR program* OR initiative* OR approach OR approaches OR model OR models OR implement* ) W/5 ( immobile OR immobility OR sedentary ) ) OR ( ( barriers OR facilitators OR increase OR improv* OR enabl* OR determin* OR evaluat* OR assess* OR track* OR monitor* OR intervention* OR "quality improvement" OR strategy OR strategies OR program* OR initiative* OR approach OR approaches OR model OR models OR implement* ) W/5 ( mobility OR movement OR mobilization OR mobilisation ) ) OR ( exercise* AND NOT ( "breathing exercise*" OR "visual* exercise*" OR "writing exercise*" ) ) OR "physical* activ*" OR ( activity W/3 level* ) OR "motor activity" OR walking OR "daily step*" OR "steps per day" OR {step count} OR {step counts} OR "step volume" OR ( ( bed OR bedside ) W/3 cycle* ) OR cycling OR bicycl* OR exergam* OR ambulation OR ambulating OR ambulate* OR locomotion ) AND TITLE-ABS-KEY ( pedomet* OR acceleromet* OR actigraph* OR activpal* OR fitbit* OR "activity-tracker*" OR "fitness tracker*" OR step-counter* OR wearable* OR "activity monitor*" OR "fitness monitor*" OR tech*-enabled OR tech*-based OR tech*-supported OR tech*-assisted OR ( technolog* W/3 ( intervention* OR implement* OR innovative OR emerging OR digital OR portable ) ) OR app OR apps OR ( application* W/3 ( phone* OR mobile OR digital OR online OR web OR electronic ) ) OR smartphone* OR "smart phone*" OR "smart watch*" OR smartwatch* OR ( ( wrist* OR wear OR worn ) W/3 ( device* OR monitor* OR sensor* ) ) OR mobile-device* OR "cell* phone" OR "mobile phone*" OR ipad* OR iphone* OR tablet OR "electronic device*" OR "handheld device*" OR "hand-held device*" OR gps OR "global positioning system*" OR sms OR "short messag* service" OR texting OR texts OR texted OR "text messag*" OR gamification OR exergam* ) AND NOT ( TITLE ( ( home-based OR outpatient* OR tele OR telephone OR telecare OR telehealth OR telemedicine OR telerehab* OR teleexercis* OR telemonitor* ) AND NOT ( inpatient* OR hospital* OR ward OR wards ) ) OR TITLE ( ( pediatric OR paediatric OR child* OR teen* OR adolescen* OR infant* OR neonat* ) AND NOT adult* ) OR KEY ( ( pediatric OR paediatric OR child* OR teen* OR adolescen* OR infant* OR neonat* ) AND NOT adult* ) OR TITLE ( pregnan* OR preoperative OR pre-operative OR prehabilitation ) |

##

## Section 4. Data Extraction Items

| **General Information** |
| --- |
| Covidence Study ID |
| Author and Year |
| Title of Article |
| Country of Intervention |
| Type of hospital inpatient setting (eg. general internal medicine, stroke etc.) |
| Funding source |
| **Intervention Details** |
| Name of intervention |
| Summary of intervention |
| Service providers who facilitated the intervention |
| Intervention coverage (unit based, program based) |
| Intervention start (days from admission) |
| Goal of intervention |
| Technology used |
| Effectiveness outcomes measured |
| Article conclusion on intervention effectiveness |
| Study limitations (as described by the article) |
| **Participant Details** |
| Number of participants (in control, treatment, other) |
| If other, please describe |
| Participant age range |
| **Implementation Details** |
| Framework used |
| Implementation plan, strategies and facilitators |
| Implementation outcomes measures (based on Proctor et al., 2011; Acceptability, Adoption, Appropriateness, Cost, Feasibility, Fidelity, Penetration, & Sustainability). |

## Section 5. Number of Included Studies Over Time (n=30).


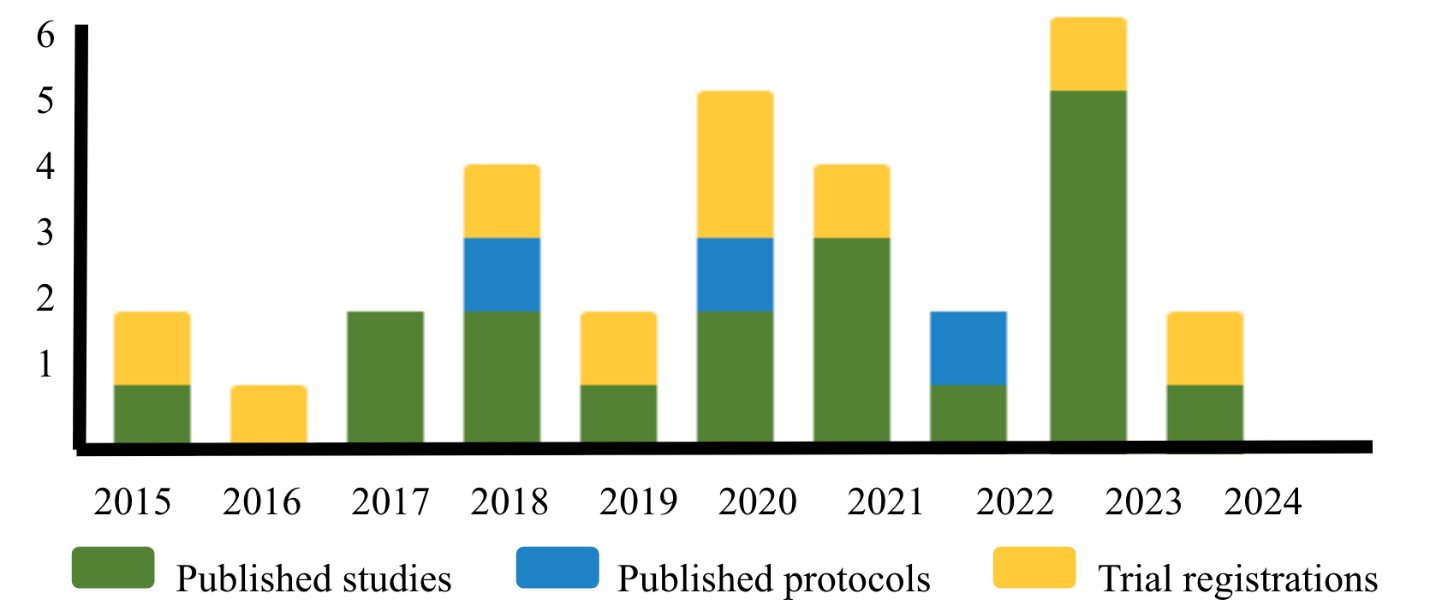


## Section 6. Complete List of Specific Technologies Used By Included Studies

| **Technology** | **Studies** |
| --- | --- |
| **Activity Trackers** | |
| Samsung Galaxy S3 Smart Watch (Samsung, Seoul, South Korea) | ACTRN12619001151123 2019 |
| Fitbit Flex ™ (Fitbit, Inc., San Francisco, CA, USA) | Conijn et al., 2020; Reed et al., 2021 |
| FT (ActiGraph GT9X Link, ActiGraph,  ProCare, Groningen, the Netherlands) | DRKS0016744 2019 |
| Fitbit One (Fitbit, Inc., San Francisco, CA, USA) | Kanai et al., 2017; Kanai et al., 2018 |
| ActivePal3 (PAL Technologies LTD, Glasgow, Scotland, UK) | ACTRN12619001151123 2019; Karlsson et al., 2023 |
| LG Lifegram, LA11M-BS Accelerometer (LG Electronics Inc., Seoul, South Korea) | No et al., 2021 |
| MOX activity monitor (Maastricht Instruments BV, Maastricht, Limburg, Netherlands) | Van Dijk-Huisman et al., 2023; Van Dijk-Huisman e al., 2020 |
| Garmin Vivofit 2 (Garmin Ltd. Olathe, KS, USA) | Van der Walt et al., 2028 |
| Physical Activity Monitor AM400 (PAM BV, Almere, Flevoland, Netherlands) | VanGrootel et al., 2023 |
| Silva Ex3 Plus Step Counter (Silva Sweden AB, Stockholm, Sweden). | Wiklund et al., 2015 |
| **Other** | |
| Google voice (Google LLC, Mountain View, CA, USA). | Ghio et al., 2022 |
| Oculus Quest 2 VR headset (Facebook Technologies LLC, Menlo Park, CA, USA) | Schrempf et al., 2023 |
| Nintendo Switch (Nintendo, Kyoto, Japan) | Takei et al., 2024 |
| Ring Fit Adventure (Nintendo, Kyoto, Japan) | Takei et al., 2024 |
| Holofit app (Holodia, Stasbourg, France) | Schrempf et al., 2023 |
| Pedatim Activity Board (Scandinavian Phystec AB) | Karlsson et al., 2023 |
| Hospital Fit (HFITAPP0, Maastricht Instruments B.V., the Netherlands) | Van Dijk-Huisman et al., 2023; Van Dijk-Huisman e al., 2020 |

Section 7. Description and examples of strategies to enable patient physical activity behavior change used by included studies.

| Strategy | Description and examples |
| --- | --- |
| Encouragement | The interventions specifically include encouragement by healthcare providers or have a designated person to help patients work through barriers to activity (personal activity coaching) |
| Collaborative goal setting | Goals were created through a discussion between the patient and a HCP, usually a physiotherapist. |
| Increasing daily goals | Activity goals increased over time. This could be a set pattern, or could depend on how much activity the patient did. |
| Progress tracking | Patients tracked their activity and either recorded it on paper or had it shown to them digitally so they could see changes over time. |
| Visual data display | How much the patient moved was displayed either to HCPs, the ward, or beside their bed so that visitors could see it. This did not include studies where the patient’s movement was displayed on the activity tracker but which would not be shared to others. |
| Patient education | Patients were educated on the importance of ambulation, typically through a discussion with a HCP and provision of educational material like brochures. |
| Environmental modification | Posters with exercises or walking routes were put up around the ward. |
| PT supported | A physiotherapist (PT) was involved in running the intervention. PTs were engaged in collaborative goal setting, encouraging patients, and educating patients. This category does not include studies where PT was performed as part of usual care. |

Section 8. Descriptions and examples of implementation strategies used by the three included studies which identified them [[15,25,41]](https://www.zotero.org/google-docs/?3rtPH3). These strategies were categorized according to the Expert Recommendations for Implementing Change [[48]](https://www.zotero.org/google-docs/?Ar1Wev).

| Strategy | Descriptions and Examples |
| --- | --- |
| Identify and prepare champions | Experienced nurses were designated as key users, and responsible for building support among colleagues, answering questions about the intervention, and incorporating the intervention into standard care. |
|  |  |
| Facilitate relay of clinical data to providers | Physical activity data was incorporated into team meetings such as multidisciplinary consultations, and successes and challenges were shared. |
|  |  |
| Conduct educational meetings | Education sessions forward staff were organized, physical therapists were trained on the intervention. |
|  |  |
| Develop educational materials | Protocols (for the intervention) for healthcare professionals were developed. |
|  |  |
| Distribute educational materials | Nurses and physicians (who would not be directly involved in the intervention) were informed that it would be happening. Protocols were distributed. |
|  |  |
