# Supplementary figures and images for: Implementing in hospital technology‐assisted mobility initiatives: A scoping review
Source: J Hosp Med. 2026 Jan 25;21(4):413–22. doi: 10.1002/jhm.70262 (PMC13064589; doi:10.1002/jhm.70262)

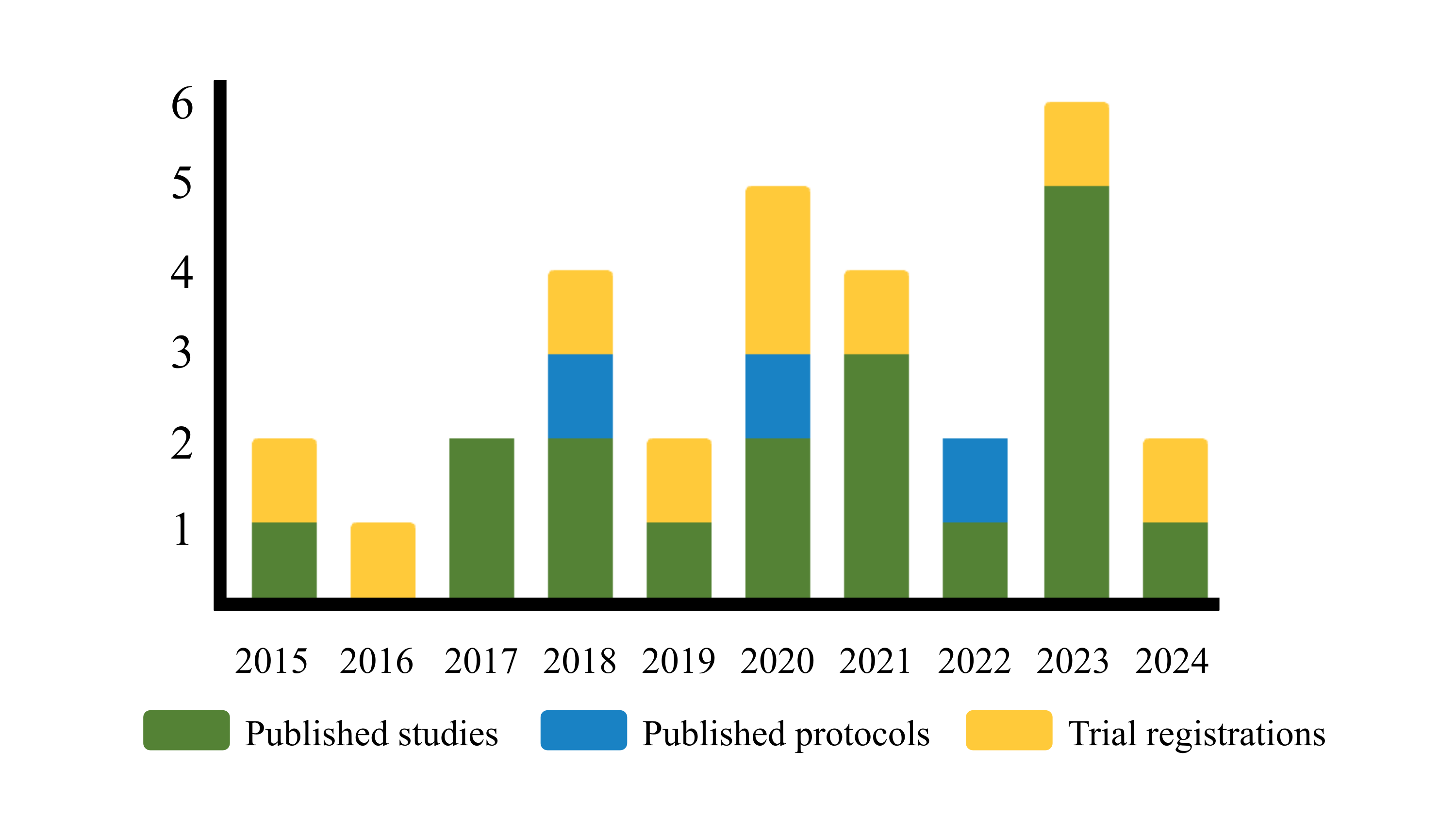

Supplement: Supplementary file 3 — Supplemental File 1‐ Figure for Section 5.tiff. [file JHM-21-413-s001.tiff]
